# Hepatic extravasation of total parenteral nutrition following umbilical venous catheter insertion: A rare complication

**DOI:** 10.1002/jpr3.12031

**Published:** 2023-12-21

**Authors:** Tal David Berger, Ayelet Gavri, Stuart Jesin, Tzipi Strauss, Batia Weiss

**Affiliations:** ^1^ Division of Pediatric Gastroenterology and Nutrition Edmond and Lily Safra Children's Hospital Tel‐Hashomer Israel; ^2^ Sackler Faculty of Medicine Tel‐Aviv University Tel‐Aviv Israel; ^3^ Neonatology Department (Neonatal Intensive Care Unit) Edmond and Lily Safra Children's Hospital Tel‐Hashomer Israel

**Keywords:** cholestasis, hepatic collections, liver injury, neonate

## Abstract

Umbilical venous catheters are commonly inserted in critically ill newborns and can lead to severe complications when misplaced. We report a preterm female with a prenatal diagnosis of duodenal atresia who presented 2 days after the surgical repair with abdominal distension, hemodynamic instability, elevated liver enzymes with severe cholestatic jaundice, and a hepatic collection on abdominal sonography. An urgent explorative laparotomy demonstrated a large amount of white, milky‐appearing fluid in the abdominal cavity. Together with the sonographic findings, this led to the diagnosis of hepatic total parenteral nutrition extravasation. Upon removal of the umbilical venous catheter line, the infant's clinical state rapidly improved; however, cholestasis continued for months, with a very slow resolution. During follow‐up, the liver enzymes normalized, and a complete resolution of the liver collection was observed, without drainage.

## 1 INTRODUCTION

Umbilical venous catheter (UVC) insertion is a common procedure used in critically ill newborns to achieve vascular access and to administer partial or total parenteral nutrition (TPN).^1^, ^2^ Yet, this procedure is not without risk, and severe complications may occur when the UVC is misplaced, such as thromboembolism, vascular injury, infection, and sepsis. In rare situations, misplacement can cause intraperitoneal extravasation of TPN followed by hepatic injury.^1^, ^2^, ^3^, ^4^, ^5^, ^6^, ^7^, ^8^, ^9^ We report a unique instance of severe liver injury caused by TPN extravasation from a UVC and review the relevant literature.

## 2 CASE PRESENTATION

We report a preterm female with a prenatal diagnosis of duodenal atresia, born at 35 weeks gestation, birth weight of 2500 g, by a spontaneous vaginal delivery.

**Figure 1:**
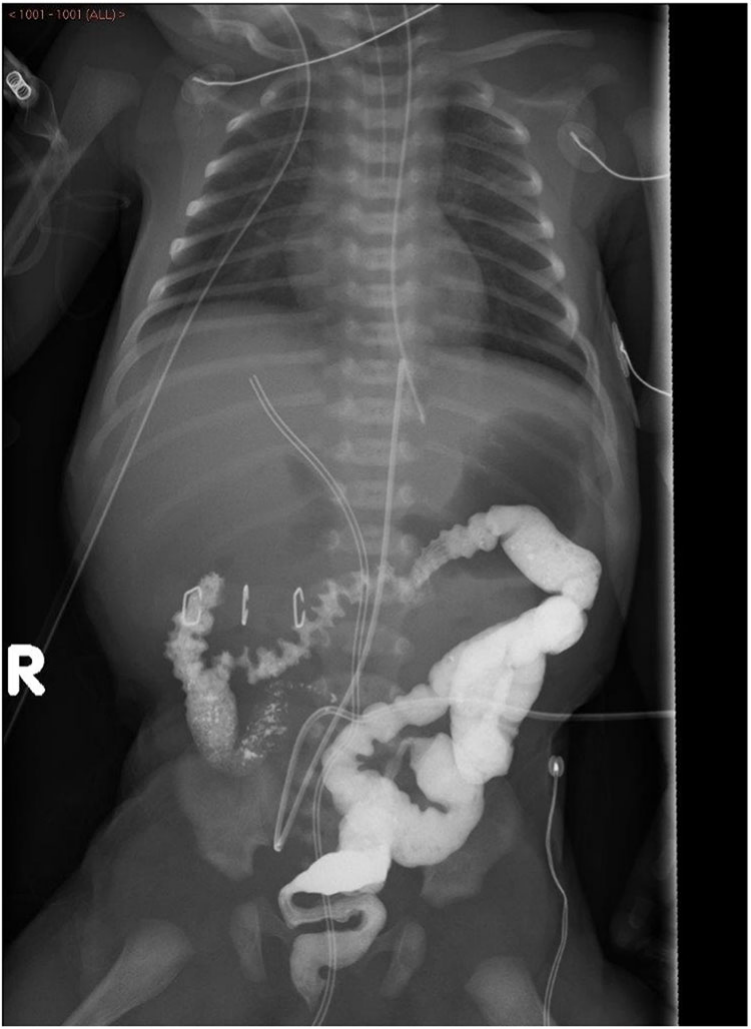
Umbilical venous and artery catheters after insertion.

On admission to the neonatal intensive care unit, the infant's vital signs and physical examination were normal. Umbilical artery and venous catheters were inserted and their locations, as verified by X‐ray, were at the T9 vertebral level, and at the level of umbilical recess and the portal vein, respectively (Figure 1). On the fourth day of life, surgery for duodenal atresia repair was performed (duodenoduodenostomy), during which the duodenum was exposed, and a transient zone between normal to dilated duodenum was identified in part 3 of the duodenum, containing a membrane inside the lumen. A diamond‐shaped anastomosis was performed with no complications. Two days after surgery, abdominal distension, respiratory distress, and hemodynamic instability appeared, raising suspicion of sepsis. Mechanical ventilation, wide‐spectrum antibiotic treatment (meropenem, vancomycin, and metronidazole), dopamine, and dobutamine were started. Laboratory tests revealed elevated liver enzymes including aspartate transaminase (AST) 796 IU/L, alanine transaminase (ALT) 428 IU/L, gamma‐glutamyl transferase (GGT) 243 IU/L, and alkaline phosphatase (ALKP) 447 IU/L; elevated bilirubin level (total 16.1 mg/dL, direct 4 mg/dL) and C‐reactive protein (up to 20.8 mg/L). No signs of intestinal perforation were observed on abdominal X‐rays.

Due to further deterioration, an urgent laparotomy was performed. Upon exploration, a large amount of white, milky‐appearing fluid was found in the abdominal cavity, with no signs of bowel ischemia or necrosis. During surgery, a large volume of fluids and additional inotropic drugs were given.

**Figure 2:**
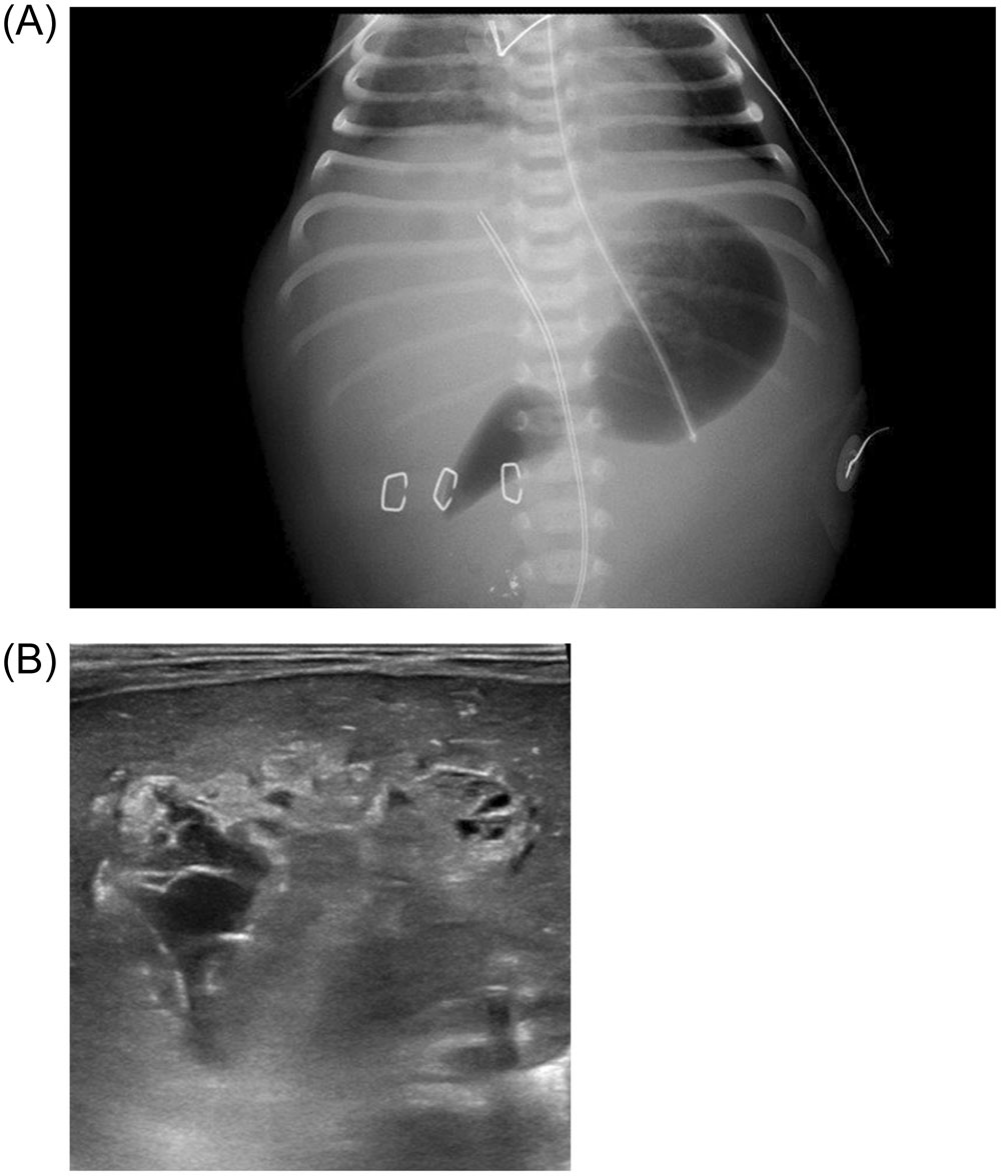
Hepatic collection findings on X‐ray (A) and sonography (B).

Several hours after surgery, acute renal failure developed with creatine levels reaching 3.13 mg/dL and urea 320 mg/dL. The liver enzyme levels further increased, up to 6343 IU/l and 2897 IU/l for AST and ALT, respectively. An abdominal X‐ray followed by an abdominal ultrasound identified two large hepatic cavities with fluid, emerging from the UVC, with dimensions of 5 × 3.4 × 3.3 cm in the liver and 1.2 × 4.1 cm in the Morrison space (Figure 2). The gallbladder and bile ducts appeared normal. TPN extravasation was suspected, and the venous line was immediately removed. The baby was treated with ursodeoxycholic acid, vitamins A, D, E, and K, and in addition, due to acute renal failure, received diuretics followed by peritoneal dialysis. During the subsequent 2 weeks, the serum direct bilirubin further increased, up to 45 mg/dL, GGT increased to 300 IU/L and ALKP to 574 IU/k, while AST and ALT normalized. Hepatic synthetic functions were normal; serum albumin was 3.5 g/dL and international normalized ratio 0.97. An attempt to drain the collection under ultrasound yielded a small amount of bloody fluid, and the procedure was consequently stopped.

During the following 2 weeks, the patient's condition slowly improved and therefore no further draining attempts were performed. The bilirubin level slowly decreased, and the hepatic collection improved in size. Renal functions gradually returned to normal. Oral feeding was successfully resumed 9 days after surgery, and the infant was discharged at age 2.5 months. At age 4 months, liver enzymes and bilirubin levels were within normal limits; and at age 14 months, no signs of collection were seen on abdominal sonography.

## 3 DISCUSSION

We describe intraperitoneal TPN extravasation after insertion of a UVC, which led to hepatic collections and severe cholestasis. To the best of our knowledge, this is the first report of severe cholestatic jaundice secondary to hepatic TPN extravasation.

Our report corroborates the case series and literature review by Grizelj et al.,^1^ in which all nine neonates with severe acute liver injury caused by UVC misplacement presented with unexplained acute deterioration. In only one neonate was the UVC tip positioned at the recommended level (between the top of the T8 vertebral body and the bottom of the T9 vertebral body) at the time of line insertion. The most prominent presenting clinical signs in that series were abdominal distention and hepatomegaly, followed by severe hypotension or anemia due to hemorrhage into cystic lesions. The diagnosis was confirmed by abdominal ultrasound in all the patients. After removal of the UVC, the liver lesions are completely or partially resolved. For six of the nine described neonates, liver enzymes recovered completely by the last follow‐up; however, in three patients, cystic lesions were still observed on ultrasonography after 4 months. One patient was lost to follow‐up, and the other two had died from unrelated medical complications.

To the best of our knowledge, only four instances were described in the literature of severe TPN extravasations into the peritoneal cavity from a misplaced UVC, leading to the need for exploratory laparotomy.^1^, ^2^, ^8^, ^9^ In all patients, the UVC line was initially in an inappropriate position. In one patient,^3^ the clinical course was similar to that of our patient, with UVC removal and exploratory laparotomy, and the hepatic collections resolved without treatment. Hagerott et al.^2^ described five neonates with extravasation of parenteral nutrition into the liver parenchyma, causing hepatic collections. In three of them, ultrasound (US)‐guided drainage of the liver collection was performed, and their condition improved; one of the lesions nearly resolved. However, most of the patients described recovered without the intervention of percutaneous drainage (as evident also in our patient). Hagerott et al. concluded that the removal of UVC and close radiologic follow‐up are the mainstay of treatment in such instances and recommended considering US‐guided drainage of hepatic lesions only for large complex lesions, failure of conservative therapy, and the presence of hemodynamic compromise. Our report demonstrates that even in such conditions, the lesion can resolve spontaneously, without drainage.

US‐guided drainage of the hepatic lesions has its own risks and may lead to bleeding, perforation, or infection, and therefore, when possible, a conservative treatment might be the preferable choice of treatment. Yet, we would still recommend considering a drainage when bacterial infection of the lesion is suspected, or when the collection is causing an obstruction of major hepatic blood vessels or bile ducts.

In conclusion, when unexplained clinical deterioration with abdominal distension presents in a neonate with a UVC, catheter‐associated liver injury should be ruled out. If rapidly treated by removal of the line, spontaneous full recovery can be expected, and liver function usually returns to normal.

## 4 CONFLICT OF INTEREST STATEMENT

Tal David Berger had made a filmed lecture for Ferring Pharmaceuticals digital library. There is no connection between the filmed lecture and this case report. The remaining authors declare no conflicts of interest.

## ETHICS STATEMENT

Informed patient consent was obtained for publication of the case details.
